# Coffee prevents fatty liver disease induced by a high-fat diet by modulating pathways of the gut–liver axis

**DOI:** 10.1017/jns.2019.10

**Published:** 2019-04-22

**Authors:** Paola Vitaglione, Giovanna Mazzone, Vincenzo Lembo, Giuseppe D'Argenio, Antonella Rossi, Maria Guido, Marcella Savoia, Federico Salomone, Ilario Mennella, Francesca De Filippis, Danilo Ercolini, Nicola Caporaso, Filomena Morisco

**Affiliations:** 1 Department of Agricultural Sciences, University of Naples ‘Federico II’, Portici, Italy; 2 Task Force on Microbiome Studies, University of Naples ‘Federico II’, Naples, Italy; 3 Department of Clinical Medicine and Surgery, Gastroenterology Unit, University of Naples ‘Federico II’, Naples, Italy; 4 Department of Medicine, University of Padua, Padua, Italy; 5 Department of Biochemistry and Medical Biotechnology, University of Naples ‘Federico II’, Naples, Italy; 6 Division of Gastroenterology, Azienda Sanitaria Provinciale di Catania, Catania, Italy

**Keywords:** Non-alcoholic steatohepatitis, Metabolic syndrome, Gut permeability, Gut microbiota, Polyphenols, ABCA1, ATP-binding cassette subfamily A1, ABCG1, ATP-binding cassette subfamily G1, ACOX1, acyl-CoA oxidase 1, ALT, alanine aminotransferase, FFAR, free fatty acid receptor, HFD, high-fat diet, HFD+COFFEE, HFD plus decaffeinated coffee, LXR-α, liver X receptor-α, NAFLD, non-alcoholic fatty liver disease, PYY, peptide YY, SD, standard diet, ZO-1, zonulin-1

## Abstract

Coffee consumption is inversely associated with the risk of non-alcoholic fatty liver disease (NAFLD). A gap in the literature still exists concerning the intestinal mechanisms that are involved in the protective effect of coffee consumption towards NAFLD. In this study, twenty-four C57BL/6J mice were divided into three groups each receiving a standard diet, a high-fat diet (HFD) or an HFD plus decaffeinated coffee (HFD+COFFEE) for 12 weeks. Coffee supplementation reduced HFD-induced liver macrovesicular steatosis (*P* < 0·01) and serum cholesterol (*P* < 0·001), alanine aminotransferase and glucose (*P* < 0·05). Accordingly, liver *PPAR- α* (*P* < 0·05) and acyl-CoA oxidase-1 (*P* < 0·05) as well as duodenal ATP-binding cassette (ABC) subfamily A1 (*ABCA1*) and subfamily G1 (*ABCG1*) (*P* < 0·05) mRNA expressions increased with coffee consumption. Compared with HFD animals, HFD+COFFEE mice had more undigested lipids in the caecal content and higher free fatty acid receptor-1 mRNA expression in the duodenum and colon. Furthermore, they showed an up-regulation of duodenal and colonic zonulin-1 (*P* < 0·05), duodenal claudin (*P* < 0·05) and duodenal peptide YY (*P* < 0·05) mRNA as well as a higher abundance of *Alcaligenaceae* in the faeces (*P* < 0·05). HFD+COFFEE mice had an energy intake comparable with HFD-fed mice but starting from the eighth intervention week they gained significantly less weight over time. Data altogether showed that coffee supplementation prevented HFD-induced NAFLD in mice by reducing hepatic fat deposition and metabolic derangement through modification of pathways underpinning liver fat oxidation, intestinal cholesterol efflux, energy metabolism and gut permeability. The hepatic and metabolic benefits induced by coffee were accompanied by changes in the gut microbiota.

Non-alcoholic fatty liver disease (NAFLD) is predominantly regarded as a hepatic manifestation of the metabolic syndrome. Approximately, 10–20 % of NAFLD cases develop non-alcoholic steatohepatitis (NASH)^(^^1^^)^, and 10–15 % of NASH cases progress to cirrhosis, which dramatically increases the incidence of hepatocellular carcinoma^(^^2^^)^. The natural history of NAFLD associated with the high incidence of obesity and type 2 diabetes makes NAFLD the most common cause of chronic liver disease in Western countries and it is predicted to become also the most frequent indication for liver transplantation by 2030^(^^3^^)^. In the last 10 years, the evidence that NAFLD actively contributes to aggravation of CVD, type 2 diabetes and chronic kidney disease became more and more robust. Therefore, a strong consensus was raised among medical doctors and researchers to consider NAFLD as a multisystem disease^(^^3^^)^. Thus, NAFLD is a worldwide health concern and medical and dietary strategies to manage and/or prevent its burden are urgent.

The aetiopathology of NAFLD involves many pathways that can be ascribed to the gut–liver axis and that regulate hepatic lipid accumulation and inflammation through the systemic metabolism, gut hormone release and the immune response. Digestion and absorption of dietary nutrients (mainly lipids and glucose), their utilisation and accumulation in the liver and in other tissues (as adipose tissue), glucose uptake and insulin sensitivity, gut microbiota composition, microbial metabolites (such as SCFA or secondary bile acids), as well as inflammatory and gut permeability status are all factors finely associated with the modulation of liver and gut health (for a detailed description of these factors, see the review by Marra & Svegliati-Baroni^(^^4^^)^).

The discovery of risk factors and mechanisms underpinning NAFLD development and progression provides biological plausibility to the epidemiological evidence showing negative association between consumption of foods and beverages rich in bioactive compounds with antioxidant and anti-inflammatory capacity (including phytochemicals and dietary fibres) and the risk of liver diseases^(^^5^^)^.

Coffee is one of the most commonly consumed beverages worldwide^(^^6^^)^ and epidemiological evidence shows that its consumption is protective towards several diseases including liver fibrosis, cirrhosis, chronic liver disease and liver cancer^(^^7^^)^. Noteworthy, inverse associations between coffee consumption and markers of liver disease risk, such as serum levels of liver enzymes, were reported for both caffeinated and decaffeinated coffee by Xiao *et al.*^(^^8^^)^, while the lack of significant associations for decaffeinated coffee in the analysis of Dickson *et al.*^(^^9^^)^ could be explained by the generally modest consumption of that type of coffee and narrow range of intakes in the population. Similarly, the recent meta-analysis by Kennedy *et al.*^(^^10^^)^, including data from the few studies that specified coffee type consumed by population, showed that the increase of caffeinated and decaffeinated coffee consumption by two cups per d reduced the risk of hepatocellular carcinoma by 27 and 14 %, respectively. These observations are in line with scientific literature showing that components in coffee other than caffeine may be critical in eliciting the health benefits of the beverage (for reviews on coffee bioactive components and mechanisms underpinning benefits, see Salomone *et al.*^(^^11^^)^, Assy *et al.*^(^^12^^)^ and Alferink *et al.*^(^^13^^)^).

Among coffee components, particular attention has been devoted to polyphenols and melanoidins. They are considered the coffee components with the highest chances of eliciting health benefits, and also against colorectal cancer, because they can arrive, in part, at the colon, thus interacting with local microbiota^(^^14^^)^. Current trends in personalised nutrition point to the microbiota as one of the principal targets to modulate health^(^^15^^)^, but it is still underinvestigated in research focused on the health effect of coffee.

We previously investigated the individual roles of coffee polyphenols and melanoidins on antioxidant and anti-inflammatory effects in the liver and blood of an animal model of NAFLD^(^^16^^)^. Data demonstrated that whole coffee was more effective than separated fractions to protect the liver from high-fat diet (HFD)-induced damage through a reduction in hepatic fat accumulation, systemic and liver oxidative stress and inflammation^(^^16^^)^.

The scientific literature indicates that gut health is implicated in the development and progression of liver diseases and very little recent evidence exists that coffee can modulate some intestinal functions^(^^17^^)^.

The present study aimed at clarifying the effect of coffee consumption on gut pathways implicated in NAFLD development such as intestinal and liver lipid metabolism, gut barrier functions and gut microbiota. To this purpose an animal study with mice fed an HFD and drinking water or a coffee extract as well as mice fed with a standard diet (SD) and drinking water for 12 weeks was implemented. Serum samples and liver histology were assessed in parallel with gene expression of molecular mediators of fat oxidation, cholesterol efflux, lipid digestion and energy metabolism regulation, gut permeability and composition of the gut microbiota.

## Materials and methods

### Animals and treatments

A sample size calculation, based on variations from a previous study^(^^18^^)^, showed that eight animals per treatment group would be sufficient to detect a threefold decrease in alanine aminotransferase (ALT) serum concentration with an α error of 0·05, a statistical power of 0·8 and two-sided testing in coffee-treated *v.* control mice.

A total of twenty-four male C57BL/6J mice, 4 weeks old, weighing 20 ± 0·5 g were housed randomly in wire-bottomed cages. Animals were obtained from Harlan Italy and were maintained under controlled temperature conditions of 22 ± 1°C, with a 12 h light–dark cycle and free access to water.

Mice were divided into three groups (*n* 8 each) and assigned to one of the following 12-week diets: (1) SD; (2) HFD; (3) HFD plus decaffeinated coffee solution (HFD+COFFEE).

The SD was a low-glycaemic control diet (TD.120455; Envigo Teklad). It contained 186 g/kg of proteins (19 % of energy), 506 g/kg of carbohydrates (51 % of energy), 62 g/kg of fat (6 % of energy), 35 g/kg of cellulose and provided 3·3 kcal/g (13·8 kJ/g).

The HFD was an adjusted-energy diet (TD.06414; Envigo Teklad). It contained 235 g/kg of protein (18 % of energy), 273 g/kg of carbohydrates (21 % of energy), 343 g/kg of fat (60 % of energy), 65·5 g/kg of cellulose and provided 5·1 kcal/g (21·3 kJ/g).

A detailed composition of the two diets is reported in Supplementary Table S1.

Coffee-containing beverages were prepared by filtering on a filter paper (Whatman grade 113; Merck KGaA), a mix of boiling water and decaffeinated coffee powder (4:1, v/w) (Illy Caffè). Filtered coffee was portioned and stored at −20°C until used. In a preliminary experiment, we found that the average daily consumption of solution (water or the coffee solution) was about 3·5 (sd 0·3) ml/mouse/d. The coffee-based beverage was prepared by diluting 1·5 ml of coffee in 100 ml of water.

The dose of administered coffee corresponded to six cups of espresso coffee or two cups of filtered coffee for a person weighing 70 kg. Food and energy intake as well as body weight were recorded weekly. Food intake was calculated based on the amount of food remaining from a known amount administered weekly.

At baseline, and at 2, 6 and 10 weeks of intervention, stool samples were collected, were snap-frozen in liquid N_2_ and stored at −80°C until DNA extraction was performed.

After 12 weeks of treatment, animals fasting for 12 h were killed by using a lethal dose of anaesthetic and the liver, duodenum, colon and caecum with all the contents were harvested and snap-frozen until analyses. A portion of liver tissues was fixed in 4 % formaldehyde and embedded in paraffin.

The experimental protocol was approved by the Ethics Committee for animal experiments at the Federico II University of Naples according to the institutional guidelines. All animals received humane care according to the criteria outlined in the National Institutes of Health Guide for the Care and Use of Laboratory Animals (National Institutes of Health Publication, eighth edition, 2011).

### Histology

Sections (5 μm thick) were obtained and stained with haematoxylin and eosin. A liver pathologist, who was blind to the intervention, performed the histological analysis, by evaluating the whole sections. Macrovesicular steatosis was assessed at low magnification (4×) as percentage of affected hepatocyte and scored as grade 0 (between 0 and 5 %), grade 1 (between 6 and 33 %), grade 2 (between 34 and 66 %) and grade 3 (>66 %). Microvesicular steatosis was also evaluated at higher magnification (20×) and recorded as percentage of affected cells. Necro-inflammatory lobular foci were sought and scored as present or absent.

### Biochemical analyses of serum samples

Serum ALT, total cholesterol and TAG were measured using the routinely automatised assays (Reflotron Plus system from Roche Diagnostic). Liver TAG content was measured in lipid extracts from previously frozen liver tissue using a TAG quantification kit (BioVision) according to the manufacturer's protocol. Data obtained were normalised by tissue protein concentration measured by the Bradford assay (Bio-Rad).

### Analysis of liver and intestinal specimens

Gene expression of *PPAR-α*, acyl-CoA oxidase 1 (*ACOX1*), ATP-binding cassette (ABC) subfamily A1 (*ABCA1*) and liver X receptor-α (*LXR-α*) in liver samples, of peptide YY (*PYY*), free fatty acid receptor-1 (*FFAR-1*), *FFAR-3*, claudin, occludin, zonulin-1 (*ZO-1*), *ABCA1* and ABC subfamily G1 (*ABCG1*) in duodenum samples and of *FFAR-1*, *FFAR-3* and *ZO-1* in colon samples was evaluated by quantitative real-time (RT)-PCR analysis. Total RNA was extracted using an RNeasy Plus Mini Kit (Qiagen) from liver, duodenum and colon biopsies. The purity of total RNA was assessed using a NanoDrop ND-100 spectrophotometer at 260 nm. A quantity of 2 μg of total RNA was used in the first-strand cDNA synthesis by TaqMan Reverse Transcription Reagents (Applied Biosystems). The cDNA was diluted with RNase-free water for a final volume of 200 ml and stored at −20°C until used. *PPAR-α, ACOX1*, *PYY*, *FFAR-1*, *FFAR-3*, claudin, occludin, *ZO-1*, *ABCA1*, *ABCG1* and *LXR-α* gene expression levels were analysed by Taq-Man Gene Expression Assays (Applied Biosystems) (Supplementary Table S2). Quantitative RT-PCR was carried out in triplicate using a pre-optimised primer/probe mixture and TaqMan Universal PCR Master Mix (Applied Biosystems) on a StepOne™ RT-PCR system in a forty-eight-well format (Applied Biosystems). The glyceraldehyde 3-phosphate dehydrogenase (*GAPDH*) housekeeping gene was used as an endogenous control, for normalisation of gene expression assays. The relative gene expression data were assessed by using the ΔΔ^Ct^ method. The sample values represent X-fold differences from a control sample (given a designated value of 1) within the same experiment.

### Lipid composition of caecal content

Extraction of lipids from caecal content samples was performed according to Gregory *et al.*^(^^19^^)^ with brief modifications. The extracts were analysed by liquid chomatography-high resolution MS by briefly adapting the method described by Bird *et al.*^(^^20^^)^. Data were acquired on an Accela U-HPLC system coupled to an Exactive Orbitrap mass spectrometer (Thermo Fisher Scientific) equipped with a heated electrospray interface. A Kinetex 2·6 µ C18 100 A column (100 × 2·1 mm) (Phenomenex) thermostated at 45°C was used. The mobile phases consisted of 40:60 water–acetonitrile, 5 mm-ammonium formate and 0·1 % formic acid (A) and 90:10 isopropanol–acetonitrile, 5 mm-ammonium formate and 0·1 % formic acid (B). A gradient elution as in Vitaglione *et al.*^(^^14^^)^ was applied. The flow rate was set at 200 µl/min and the injection volume was 10 µl. Acquisition was performed in both positive and negative ionisation modes, in the mass range of *m*/*z* 120–1200. The resolving power was set to 50 000 full width at half-maximum (*m*/*z* 200) resulting in a scan time of 1 s. The automatic gain control was used in balanced mode (1 × 106 ions); maximum injection time was 100 ms. The interface parameters were as follows: spray voltage was at 3·5 kV (positive mode) and 3·0 kV (negative mode), capillary voltage 30 V, heater temperature at 350°C, capillary temperature at 250°C, sheath gas at 35 arbitrary units, and auxiliary gas at 15 arbitrary units. Chromatographic data acquisition and peak integration were performed using Xcalibur software (Thermo Fisher Scientific).

NEFA identification was obtained using ExactFinder 2.0 software (Thermo Fisher Scientific) by the application of a homemade database. Specific molecular formulas and their respective *m*/*z* ratios were included in ExactFinder and the following parameters were selected: exact mass of monoisotopic ion (experimental mass tolerance accuracy less than 5 parts per million), isotopic pattern, retention time, signal:noise ratio higher than 5.

TAG were detected in positive mode as predominately (M + NH_4_)^+^ ions and were integrated in the chromatographic region between retention times of 22–24·5 min. NEFA were detected in negative mode as (M − H)^−^ ions in the chromatographic region between 3 and 7·5 min.

### Gut microbiota composition

Faecal samples (about 0·3 g) were used for DNA extraction with the PowerSoil DNA Isolation kit (Mo Bio Laboratories, Inc.). The V1–V3 region of the 16S rRNA gene (about 520 bp) was amplified and PCR libraries were prepared and sequenced as previously reported^(^^21^^)^.

The sequences were analysed and filtered using QIIME 1.9.0 software^(^^22^^)^. Quality filtering, *de novo* operational taxonomic unit (OTU) picking and taxonomy assignment were carried out as previously described^(^^23^^)^. To avoid biases due to the different sequencing depths, OTU tables were rarefied to the lowest number of sequences per sample.

The 16S rRNA gene sequences produced in this study are available at the Sequence Read Archive (SRA) of the National Center for Biotechnology Information (NCBI), under accession number PRJNA434511.

### Statistical analysis

All the animals (eight per group) were included in each analysis. Data are reported as means with their standard errors. Groups were compared using ANOVA followed by Tukey's multiple-comparison tests. A *P* value of less than 0·05 was considered statistically significant.

For the faecal microbiota composition statistical analyses were carried out using R (https://www.r-project.org). Differential abundance of specific taxa according to diet or coffee assumption was determined by DeSeq2^(^^24^^)^. Principal components analysis was carried out on the normalised abundance (log_10_) of the microbiota at genus level (*dudi.pca* function in the R package *made4*). Permutational multivariate ANOVA (non-parametric MANOVA) based on Jaccard distance matrix was carried out by using 999 permutations to detect significant differences in the overall bacterial community composition as affected by the type of diet or coffee consumption, by using the *adonis* function in the *vegan* package.

## Results

### Body weight and food intake

After 12 weeks, HFD-fed mice (with or without coffee) weighed significantly more than mice fed the SD (*P* < 0·001 for both groups) (Supplementary Fig. S1). Starting from week 8, HFD- and HFD+COFFEE-fed mice had different growth curves. HFD+COFFEE-fed mice gained progressively less body weight compared with HFD-fed mice (*P* < 0·05). This difference was maintained until week 12 when HFD+COFFEE-fed mice weighed 34·8 (se 2·5) g and HFD-fed mice weighed 38·4 (se 3·0) g (*P* < 0·01). Food intake data showed that HFD- and HFD+COFFEE-fed mice had higher energy intake than SD-fed animals (386·2 (se 7·1) and 369·2 (se 5·8) *v.* 253·0 (se 2·6) kcal (1615·9 (se 29·7) and 1544·7 (se 24·3) *v.* 1058·6 (se 10·9) kJ); *P* < 0·001 for both comparisons). Moreover, HFD+COFFEE-fed mice had higher food intake than HFD-fed mice (386·2 (se 7·1) *v.* 369·2 (se 5·8) kcal (1615·9 (se 29·7) *v.* 1544·7 (se 24·3) kJ); *P* < 0·05) (Supplementary Fig. S1(B)).

As concerns liver weight, data showed a reduced liver:body weight ratio in HFD+COFFEE-fed mice compared with HFD-fed mice (3·06 (se 0·06) *v.* 3·47 (se 0·15); *P* < 0·05) and SD-fed mice (3·06 (se 0·06) *v.* 3·59 (se 0·06); *P* < 0·01).

### Serum parameters and liver steatosis

Serum total cholesterol, TAG, ALT and glycaemia are reported in Table 1. Both groups of HFD-fed mice had higher circulating concentrations of total cholesterol than SD-fed mice (*P* < 0·0001 for HFD and *P* = 0·0097 for HFD+COFFEE *v.* SD). However, lower concentrations were recorded in HFD+COFFEE-fed than HFD-fed mice (*P* = 0·0001). Similarly, coffee intake reduced serum ALT in HFD-fed mice (*P* = 0·0280). Interestingly, a significant reduction in blood glucose in HFD+COFFEE- *v*. SD-fed mice (*P* = 0·0085) was also observed.

**Table 1.** Serum levels of total cholesterol, TAG, alanine aminotransferase (ALT) and glucose (Mean values with their standard errors; eight mice per group)

	SD		HFD		HFD+COFFEE	
	Mean	sem	Mean	sem	Mean	sem
Total cholesterol (mg/dl)‡	106·83	11·32	238·50**	42·30	160·63**††	23·79
TAG (mg/dl)‡	103·67	22·17	110·38	34·72	110·63	20·39
ALT (U/l)	53·33	39·82	56·88	16·07	45·86†	36·20
Glucose (mg/dl)‡	271·9	4·92	275·5	6·07	82·0**††	7·07

SD, standard diet; HFD, high-fat diet; HFD+COFFEE, HFD plus decaffeinated coffee.

** Mean value was significantly different from that for the SD-fed mice (*P* < 0·001; ANOVA and Tukey's *post hoc* analysis).

Mean value was significantly different from that for the HFD-fed mice: † *P* < 0·05, †† *P* < 0·001 (ANOVA and Tukey's *post hoc* analysis).

‡ To convert cholesterol in mg/dl to mmol/l, multiply by 0·0259. To convert TAG in mg/dl to mmol/l, multiply by 0·0113. To convert glucose in mg/dl to mmol/l, multiply by 0·0555.

**Fig. 1.**
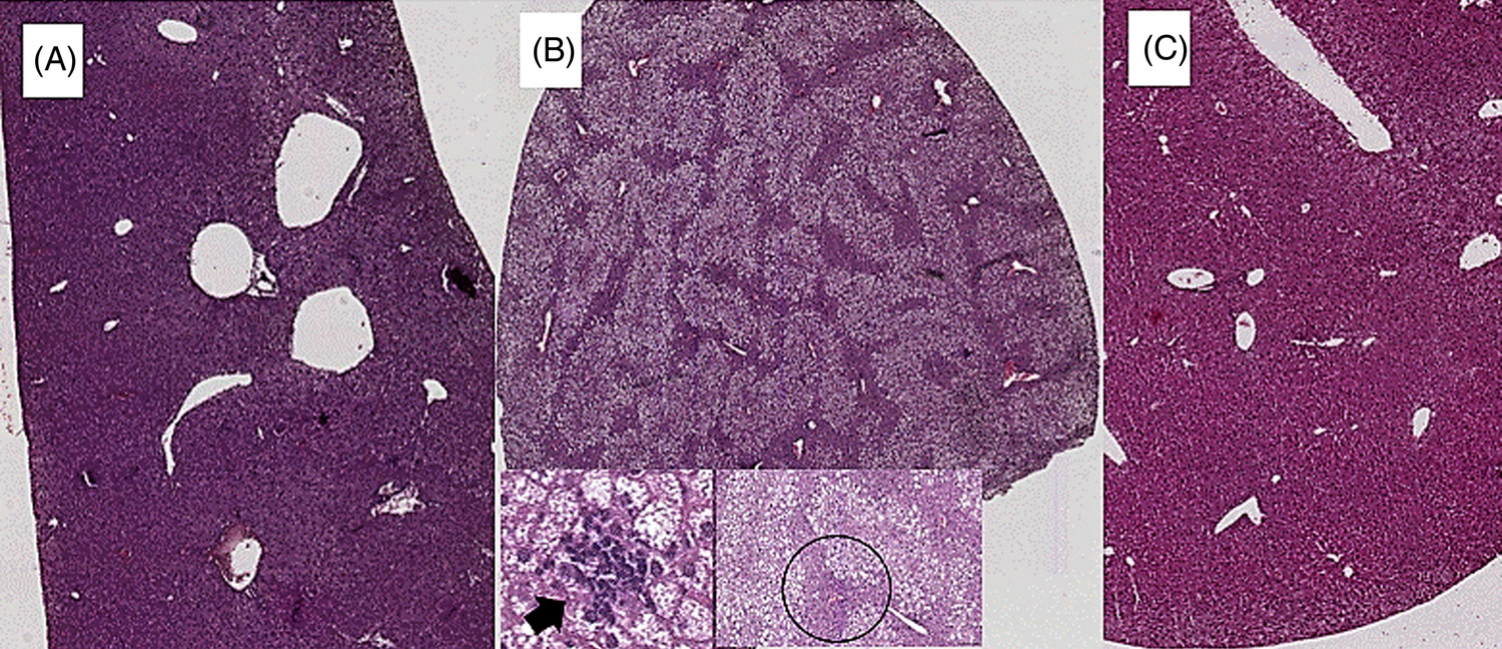
Liver histology. (A) Liver from a mouse fed a standard diet showing complete absence of steatosis. (B) Severe steatosis in mouse fed a high-fat diet (HFD), only sparing a small rim of peri-portal hepatocytes (black circle); lobular necro-inflammatory foci were seen in this liver (→). (C) Absence of steatosis in an HFD plus decaffeinated coffee-fed mouse.

Coffee consumption caused a reduction in liver steatosis induced by the HFD. Macrovesicular steatosis was scored 0 in all livers from SD- and HFD+COFFEE-fed mice, whereas in the HFD group only three cases had a score of 0. The difference in steatosis grade between the HFD and SD and HFD+COFFEE groups was significant (*P* < 0·01) (Supplementary Fig. S2). Microvesicular steatosis was also less severe in HFD+COFFEE- than HFD-fed mice, even if the difference between the two groups was not statistically significant. Necro-inflammatory lobular foci were seen in four of the HFD-fed mice and none of the HFD+COFFEE-fed group (*P* = 0·01) (Fig. 1).

### Liver factors underpinning fat deposition

**Fig. 2.**
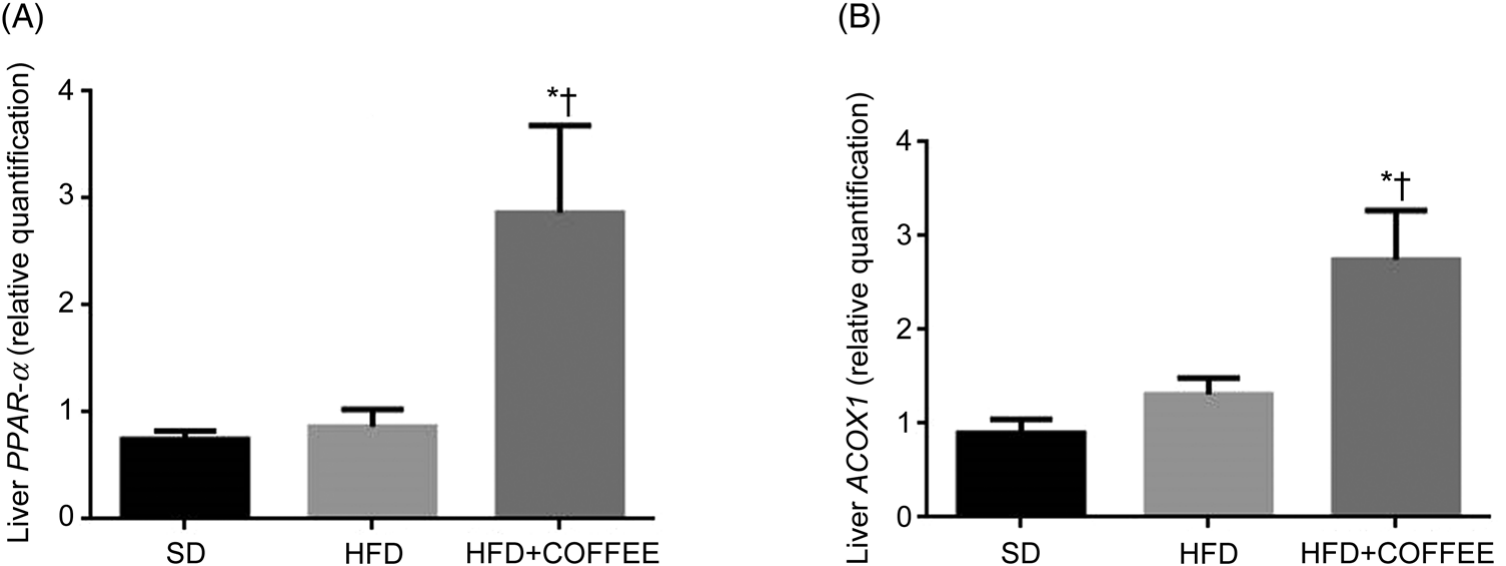
Modulators of fat oxidation in the liver. Liver *PPAR-α* (A) and acyl-CoA oxidase 1 (*ACOX1*) (B) gene expression in standard diet (SD), high-fat diet (HFD) and HFD plus decaffeinated coffee (HFD+COFFEE) mice. Data are means, with standard errors represented by vertical bars. * Mean value was significantly different from that for the SD-fed mice (*P* < 0·05; ANOVA and Tukey's *post hoc* analysis). † Mean value was significantly different from that for the HFD-fed mice (*P* < 0·05; ANOVA and Tukey's *post hoc* analysis).

Fig. 2 shows the gene expression of liver *PPAR-α* and *ACOX1* of mice in the three groups. Data showed a threefold and twofold up-regulation of *PPAR-α* and *ACOX1* mRNA expression, respectively, in the liver of HFD+COFFEE-fed mice compared with SD-fed animals (*P* = 0·0228 and *P* = 0·0103) and with HFD-fed mice (*P* = 0·0160 and *P* = 0·0355). This suggested that coffee can promote β-oxidation in the liver.

### Liver and intestinal factors underpinning cholesterol efflux

**Fig. 3.**
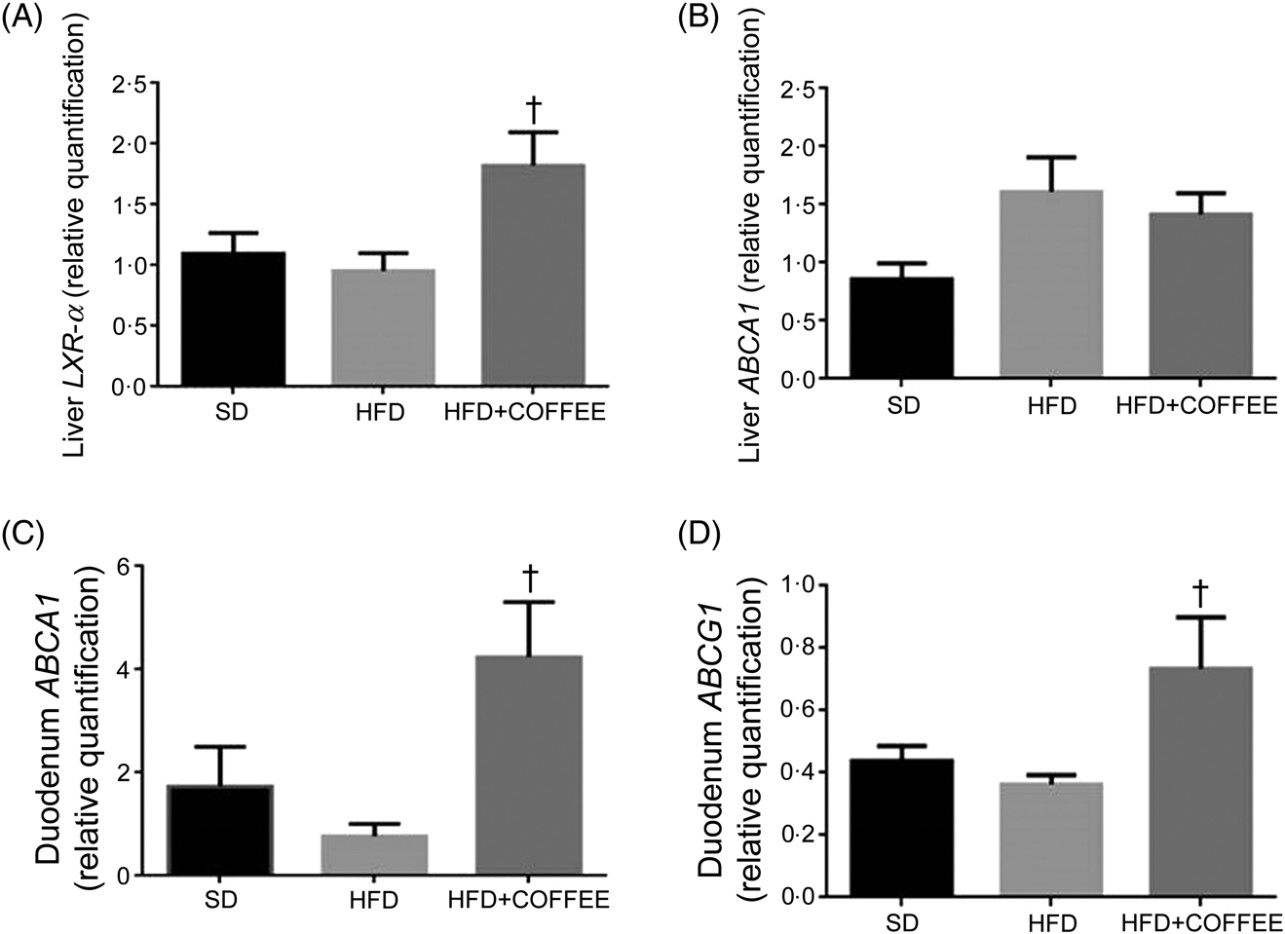
Modulators of liver and intestinal cholesterol efflux. Gene expression of liver X receptor-α (*LXR-α*) (A) and ATP-binding cassette subfamily A1 (*ABCA1*) (B) in the liver and of *ABCA1* (C) and ATP-binding cassette subfamily G1 (*ABCG1*) (D) in the duodenum. Data are means, with standard errors represented by vertical bars. † Mean value was significantly different from that for the HFD-fed mice (*P* < 0·05; ANOVA and Tukey's *post hoc* analysis).

Fig. 3 shows the effect of treatments on gene expression of hepatic *LXR-α* (Fig. 3(A)) and *ABCA1* (Fig. 3(B)) as well as duodenal *ABCA1* (Fig. 3(C)) and *ABCG1* (Fig. 3(D)). Liver *LXR-α* mRNA expression was increased by coffee consumption, while liver *ABCA1* mRNA expression appeared slightly increased following the HFD compared with the SD but the difference did not reach statistical significance (*P* = 0·07). On the other hand, at intestinal level, an up-regulation of both *ABCA1* and *ABCG1* gene expression in HFD+COFFEE-fed *v*. HFD-fed mice (*P* = 0·0164 and *P* = 0·0306) was found; however, although the mRNA expressions were slightly increased *v*. SD-fed mice, they were not significant (*P* = 0·0882 and *P* = 0·0895). These findings suggested that coffee intake can stimulate cholesterol efflux.

### Intestinal factors implicated in lipid sensing and energy metabolism

**Fig. 4.**
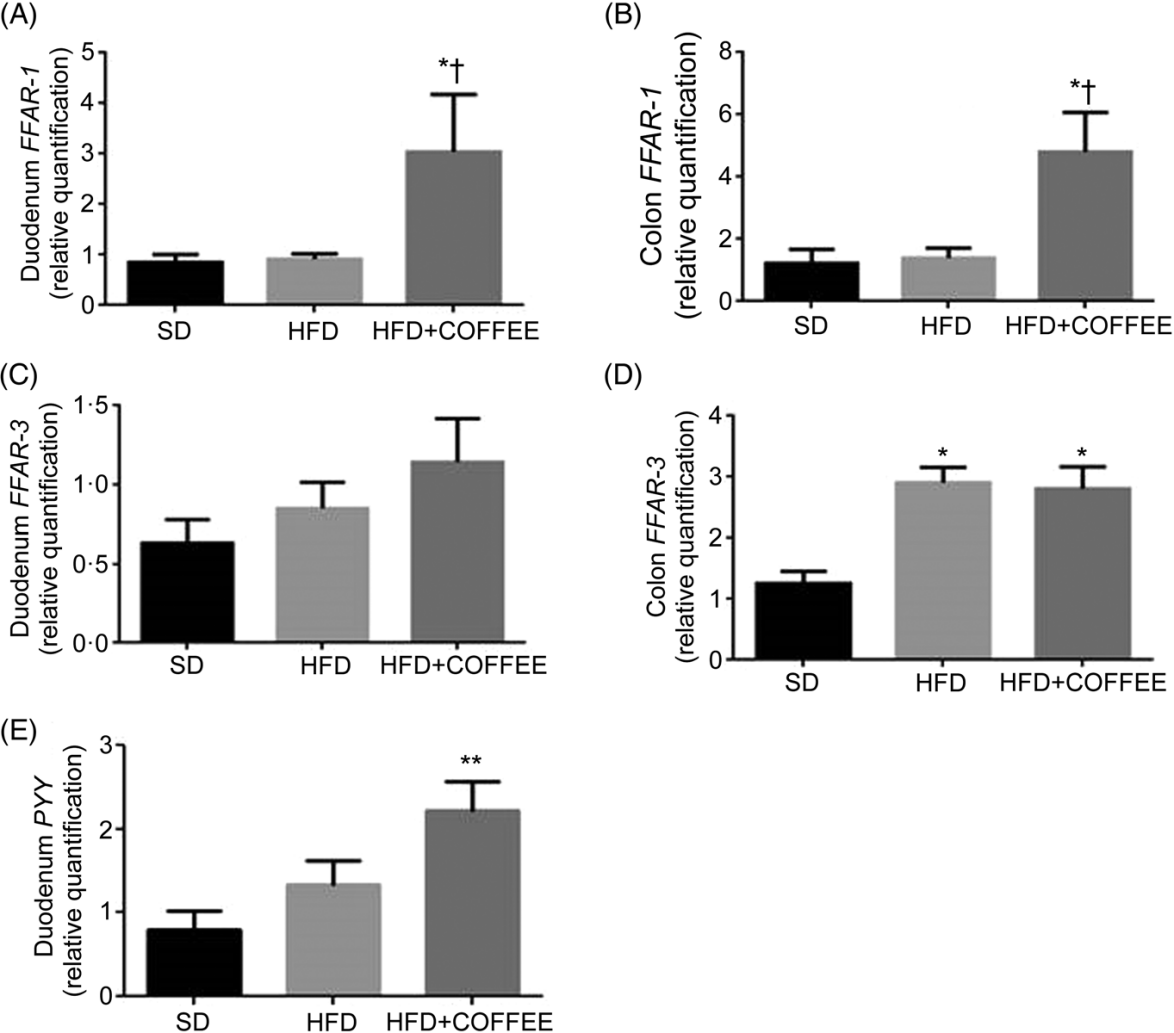
Modulators of intestinal lipid sensing and energy metabolism. Gene expression of free fatty acid receptor-1 (*FFAR-1*) (A and B) and *FFAR-3* (C and D) in the duodenum and colon, and peptide YY (*PYY*) (E) in the duodenum of standard diet (SD), high-fat diet (HFD) and HFD plus decaffeinated coffee (HFD+COFFEE) mice. Data are means, with standard errors represented by vertical bars. Mean value was significantly different from that for the SD-fed mice: * *P* < 0·05, ** *P* < 0·01 (ANOVA and Tukey's *post hoc* analysis). † Mean value was significantly different from that for the HFD-fed mice (*P* < 0·05; ANOVA and Tukey's *post hoc* analysis).

Fig. 4 shows the effect of treatments on gene expression of duodenal and colonic *FFAR-1* (Fig. 4(A) and (B)) and *FFAR-3* (Fig. 4(C) and (D)) as well as duodenal *PYY* (Fig. 4(E)). Coffee consumption up-regulated *FFAR-1* in both duodenum (*P* = 0·021 *v*. HFD and *P* = 0·029 *v*. SD) and colon tissue (*P* = 0·031 *v*. HFD and *P* = 0·043 *v*. SD) whereas *FFAR-3* in the colon was up-regulated by the HFD independently from coffee (*P* = 0·005 for HFD *v*. SD and *P* = 0·012 for HFD+COFFEE *v*. SD). *PYY* gene expression was up-regulated in the duodenum of HFD+COFFEE- *v.* SD-fed mice (*P* *<* 0·01) while no differences were recorded between HFD+COFFEE- and HFD-fed mice or HFD- and SD-fed mice.

Interestingly, the analysis of lipids in the caecum content showed that HFD+COFFEE-fed mice had 2·2 and 2·5 times higher amount of total lipids in the caecum than HFD- and SD-fed mice, respectively. Furthermore, looking at lipid composition, HFD+COFFEE-fed mice showed a NEFA:TAG ratio that was ten times lower than HFD-fed mice (0·02 *v*. 0·16 %), thus meaning that the lipids were mainly in the form of TAG (Supplementary Fig. S3).

### Intestinal permeability and gut microbiota

**Fig. 5.**
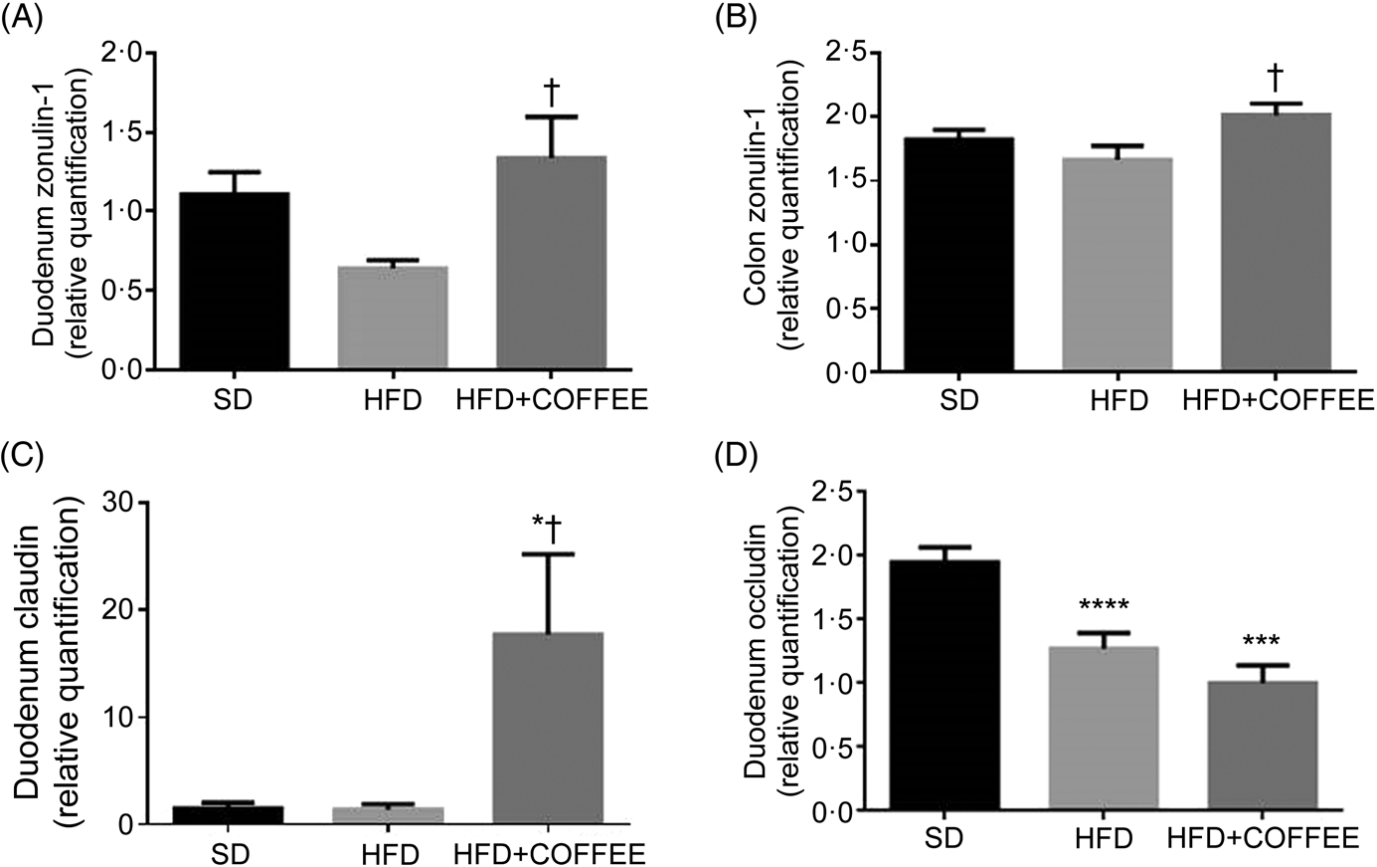
Modulators of intestinal permeability. Gene expression of the tight junction proteins zonulin-1 in the duodenum (A) and colon (B), and claudin (C) and occludin (D) in the duodenum of standard diet (SD), high-fat diet (HFD) and HFD plus decaffeinated coffee (HFD+COFFEE) mice. Data are means, with standard errors represented by vertical bars. Mean value was significantly different from that for the SD-fed mice: * *P* < 0·05, *** *P* < 0·005, **** *P* < 0·001 (ANOVA and Tukey's *post hoc* analysis). † Mean value was significantly different from that for the HFD-fed mice (*P* < 0·05; ANOVA and Tukey's *post hoc* analysis).

To establish whether coffee consumption may modulate intestinal permeability, the gene expression of the tight junctions *ZO-1*, occludin and claudin on intestinal specimens was assessed (Fig. 5). Significant up-regulations of duodenal and colonic *ZO-1* and duodenal claudin mRNA expression with the HFD+COFFEE *v*. HFD (*P* = 0·035, *P* = 0·044 and *P* = 0·016, respectively) and *v*. the SD (*P* = 0·020) were found.

Conversely, occludin gene expression was down-regulated by the HFD and HFD+COFFEE *v*. the SD (*P* = 0·0027 and *P* = 0·0006, respectively).

**Fig. 6.**
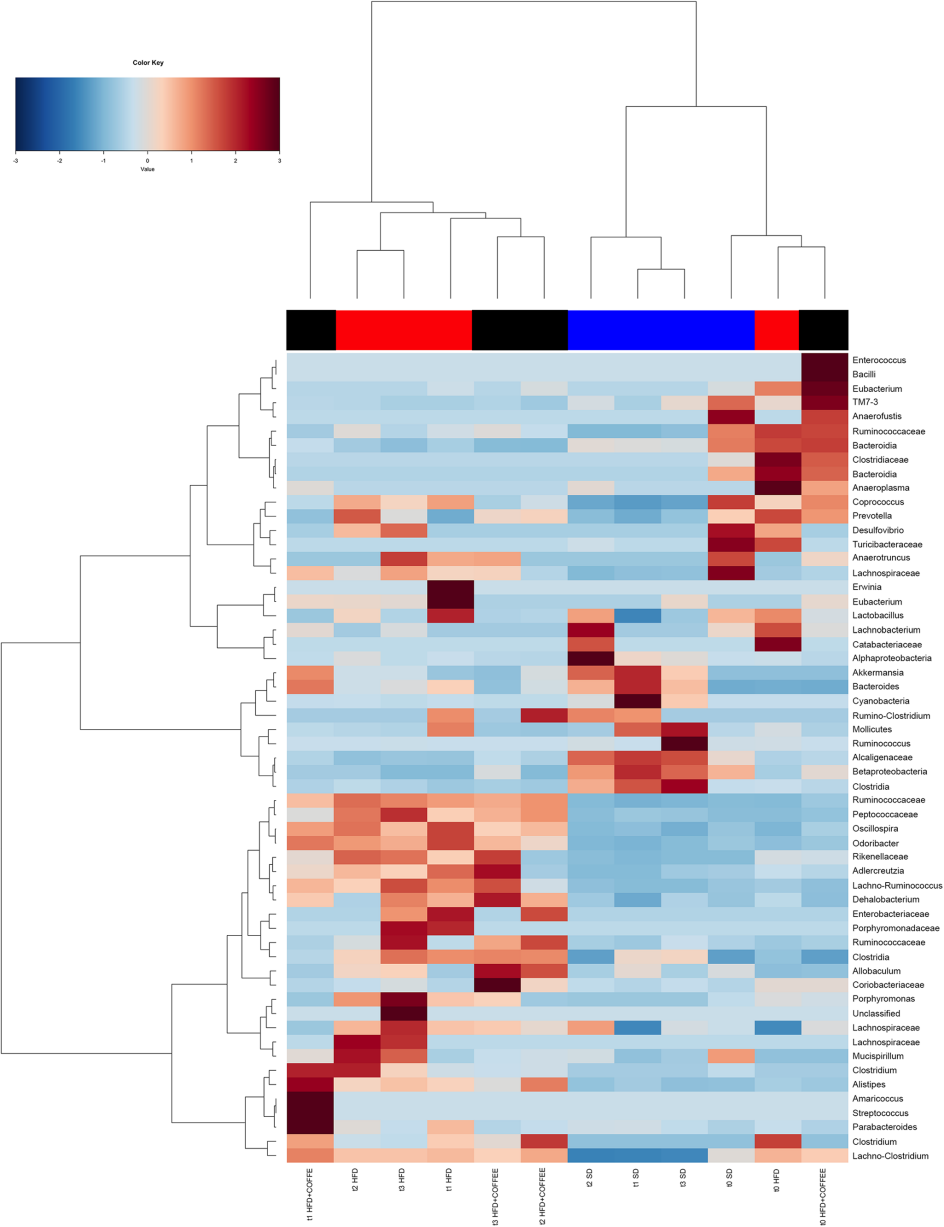
Gut microbiota. Hierarchical Ward's linkage clustering of the samples based on the Pearson's correlation coefficient for the abundance of taxa present in at least 10 % of the samples. The colour scale indicates the scaled abundance of each variable, denoted as the *Z*-score: red, high abundance; blue, low abundance. Column bars are coloured according to the dietary treatment: blue, standard diet (SD)-fed mice; red, mice fed a high-fat diet (HFD); black, mice fed an HFD and decaffeinated coffee (HFD+COFFEE). Samples are coded according to the type of diet (SD or HFD) and the length of treatment (t0, baseline; t1, 2 weeks; t2, 6 weeks; t3, 10 weeks).

Faecal microbiota was strongly affected by the type of diet (HFD *v*. SD) (*P* < 0·05). In particular, levels of *Alistipes*, *Odoribacter* and *Allobaculum* increased while *Muribaculum* was reduced with the HFD compared with the SD (*P* < 0·05) (Fig. 6). Interestingly, coffee intake was associated with a significant increase in *Alcaligenaceae* in HFD-fed mice (0·33 % in HFD+COFFEE *v*. 0·03 % in HFD), regardless of the length of the treatment. Principal components analysis showed a clear clustering of the samples according to diet (Supplementary Fig. S4(A)). Nevertheless, when the analysis was carried out on HFD- or SD-fed mice separately, an effect of coffee intake was observed. Samples after 6 and 10 weeks of coffee consumption (HFD+COFFEE; Supplementary Fig. S4(B)) separated from the HFD. This separation was mainly driven by the different abundance of *Alistipes*, *Bacteroides* and *Odoribacter.*

## Discussion

**Fig. 7.**
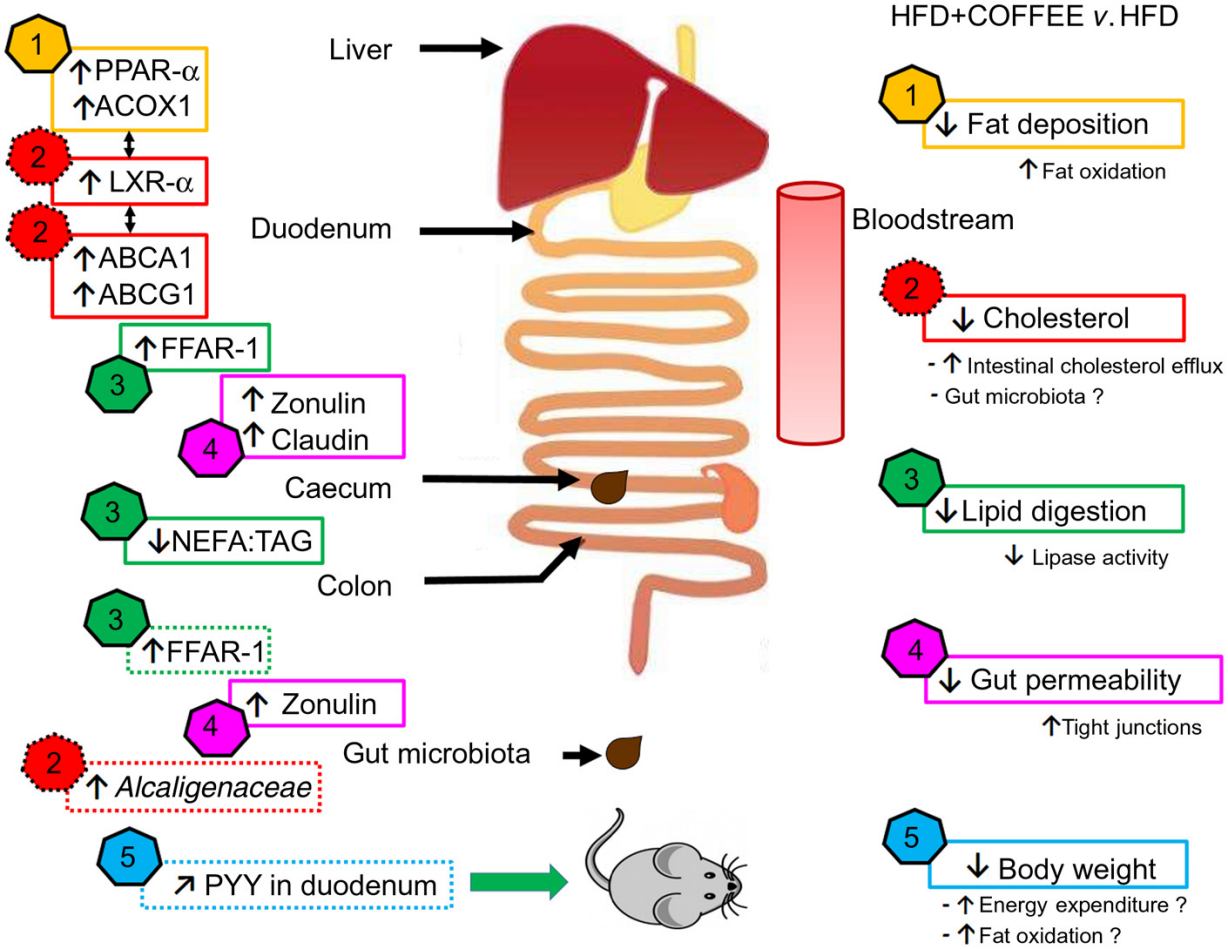
Schematic representation of the main findings of the study. In the right side of the scheme, five effects of coffee consumption and the demonstrated or hypothesised (if followed by a ‘?’) mechanisms underpinning those effects are listed. In the left side of the scheme, pathways involved at specific sites of the gastrointestinal tract are reported (↑, increase; ↓, reduction; ↗, slight increase but not significant). The data showed that coffee consumption determined: (1) reduction in hepatic fat deposition by increasing fat oxidation in the liver as demonstrated by up-regulation of *PPAR-α* and acyl-CoA oxidase 1 (*ACOX1*) gene expression; (2) reduction in circulating cholesterol by activating cholesterol intestinal efflux through up-regulation of gene expressions of liver X receptor-α (*LXR-α*) in the liver and intestinal ATP-binding cassette subfamily A1 (*ABCA1)* and ATP-binding cassette subfamily G1 (*ABCG1*). This effect was possibly sustained by the increased abundance of *Alcaligenaceae* in gut microbiota; (3) reduction in lipid digestion and amelioration of the intestinal system involved in lipid sensing as demonstrated by the decreased ratio of NEFA:TAG found in the caecum content of coffee-drinking mice and by up-regulation of free fatty acid receptor-1 (*FFAR-1*) mRNA expression in duodenum and colon; (4) reduction in gut permeability through a restoration of tight junction proteins in the duodenum and colon as demonstrated by up-regulation of zonulin-1 and claudin gene expression; (5) control of body weight possibly through increase in energy expenditure and fat oxidation. Those conditions might be induced by an improvement in lipid sensing that possibly influenced energy metabolism regulation through amelioration of insulin sensitivity and a negligible up-regulation of intestinal peptide YY (*PYY*) gene expression.

In this study, the molecular mechanisms underpinning the effects of coffee on NAFLD induced by an HFD were elucidated by focusing on pathophysiological pathways linking the gut and liver. The main findings are summarised in Fig. 7.

Coffee consumption reduced HFD-induced liver steatosis and circulating ALT, which is consistent with our previous findings^(^^16^^)^. The reduced fat deposition (as demonstrated by histopathology and supported by the reduced liver:body weight ratio) and inflammation in the liver could be a consequence of an increased fat oxidation^(^^25^^)^, which was supported by the up-regulation of PPAR-α. This is in agreement with other studies demonstrating the ability of polyphenols, as pure compounds or as part of foods, to induce lipid oxidation in the liver of animals fed with an HFD^(^^26^^)^. The hypothesis of coffee-induced fat oxidation in the liver is further supported, in the present study, by the up-regulation of ACOX1. Indeed, ACOX1 is responsible for the second reaction of peroxisomal β-oxidation^(^^27^^)^ and its up-regulation together with PPAR-α was a key factor in the hypolipidaemic effect of a polyphenol-rich walnut extract^(^^28^^)^ as well as of an apple peel extract and quercetin in HFD-fed mice^(^^29^^)^.

Differently from our previous study in rats^(^^16^^)^ coffee did not modulate serum TAG but reduced blood cholesterol and glucose in the present study. Both effects are possibly mediated by liver LXR-α whose activation is in line with that of PPARα^(^^30^^)^. LXR-α could regulate systemic cholesterol homoeostasis by increasing biliary cholesterol excretion through regulation of the intestinal membrane transporters ABCA1 and ABCG1^(^^31^^)^. Indeed, the two transporters act synergistically to remove cholesterol from cells: ABCA1 converts lipid-poor apoA-I to partially lipidated ‘nascent’ lipoproteins which are effective acceptors for cholesterol exported through the basolateral membrane by ABCG1^(^^32^^)^. On the other hand, activation of LXR-α could also modulate liver gluconeogenesis and adipose tissue insulin-sensitive glucose transporter (GLUT4) by inducing glucose utilisation in the liver and storage in adipocytes^(^^33^^)^. At the intestinal level, the up-regulation of gene expression of the long-chain fatty acids receptor FFAR-1 could further support the ameliorated glucose metabolism in coffee- compared with water-drinking mice. FFAR-1 activation by NEFA in the intestinal lumen induces the secretion of glucagon-like peptide-1 and glucose-dependent insulinotropic polypeptide, which can stimulate insulin secretion glucose-dependently, thus possibly contributing to the hypoglycaemic effect elicited by coffee in HFD-fed mice^(^^34^^)^. Moreover, FFAR-1 activation elicits cholecystokinin response that, in turn, can stimulate secretion of pancreatic lipase and fat absorption^(^^35^^)^. Therefore, it was hypothesised that FFAR-1 up-regulation was an intestinal adaptive mechanism to compensate for the lacking fat digestion caused by the inhibitory activity of coffee polyphenols on pancreatic lipase^(^^36^^–^^38^^)^. Indeed, lipid characterisation of caecum contents showed that the NEFA:TAG ratio was lower in HFD+COFFEE-fed mice than HFD-fed mice. An ameliorated fat absorption consequent to FFAR-1 up-regulation and possibly induced by a reinforced cholecystokinin response might provide an explanation for the similar TAG serum level in HFD- and HFD+COFFEE-fed mice despite the latter showing a reduced fat digestion.

Although statistical significance was not reached, coffee consumption slightly increased the gene expression of duodenal *PYY* in HFD+COFFEE-fed mice. This observation goes in parallel with a higher food intake but lower body weight gain for HFD+COFFEE- *v*. HFD-fed mice. We hypothesised that possibly higher levels of PYY in these mice failed to increase satiety because in the presence of lipase inhibitors from coffee an attenuation of the inhibitory effect of oral fat on food intake might have occurred^(^^39^^)^. On the other hand, the lower body weight of coffee-drinking mice than the others lets us hypothesise that higher levels of PYY might have boosted energy expenditure and fat oxidation rate in HFD+COFFEE-fed mice^(^^40^^)^.

Another important finding of the present study is that coffee might have ameliorated gut permeability as suggested by the restoration of zonulin-1 gene expression in the duodenum and colon as well as claudin in the duodenum of HFD+COFFEE-fed mice *v.* HFD-fed mice. An improved gut barrier function induced by coffee in the present study could explain the reduced liver steatosis and the lower serum levels of cholesterol and glucose. Moreover, it is in agreement with our previous observation in rats^(^^41^^)^ and with the recent findings of Brandt *et al.*^(^^17^^)^ who found that consumption of decaffeinated coffee in mice fed with a high-fat, -fructose and -cholesterol diet increased intestinal protein expression of zonulin-1 and occludin, reduced bacterial endotoxin concentration in portal blood and mRNA expression of lipopolysaccharide-binding protein in the liver compared with the mice fed with the same diet without coffee. It is well known that HFD is associated with dysbiosis, intestinal mucosa inflammation and a leaky gut condition leading to metabolic endotoxaemia and consequent liver fat accumulation and inflammation^(^^42^^)^. Prebiotics, such as inulin-type fructans, were shown to revert those conditions by modulating gut microbiota^(^^43^^–^^45^^)^. However, in the present study the overall structure of the gut microbiota was not significantly changed by coffee consumption, as determined by MANOVA. Nevertheless, increased abundance of *Alcaligenaceae* was found in mice fed the HFD and drinking coffee. *Alcaligenaceae* were shown to metabolise oestradiol *in vitro*^(^^46^^)^ and were negatively associated with the total cholesterol concentration in a population with atherosclerosis^(^^47^^)^. Therefore, it can be speculated that these bacteria might be implicated in the intestinal re-absorption of cholesterol, thus providing an upstream mechanism of cholesterol efflux to the effect of coffee on lowering serum cholesterol in the present study. A high abundance of *Alcaligenaceae* was also observed by Zhou *et al.*^(^^48^^)^ in mice supplemented with whole grain oat (WGO) flour and experiencing a lower blood cholesterol and improvement of insulin sensitivity in comparison with the mice supplemented with low bran oat (LBO) flour. The similar findings between the present study and the study by Zhou *et al.*^(^^48^^)^ could be related to the feature of WGO and coffee to be sources of antioxidant dietary fibres. Indeed, the WGO diet contained a 1·4 % (w/w) more soluble fibre (mainly β-glucans) and specific phytochemicals including phenolic acids, alkyl- and alkenylresorcinols, and avenanthramides compared with LBO. Coffee used in the present study contained melanoidins and polyphenols. Melanoidins are known to behave physiologically as dietary fibre, i.e. they can arrive undigested to the colon and can be fermented by local microbiota^(^^49^^)^. In addition, since melanoidins entrap polyphenols in their structure, they are chemically similar to the antioxidant cereal dietary fibres and, similarly, they behave as natural carriers of polyphenols to the colon, thus possibly beneficing colon health and nutrient metabolism through the modulation of the gut microbiota composition and functions^(^^14^^,^^50^^)^.

Altogether, the mechanisms showed in our study support epidemiological data demonstrating that coffee reduces the prevalence and incidence of the metabolic syndrome^(^^51^^)^.

In conclusion, we provided first evidence that coffee consumption reduces HFD-induced liver damage by modulating pathways involved in the gut–liver axis.

Coffee reduced the HFD-induced liver steatosis and metabolic derangement by:

Reducing hepatic fat deposition possibly through increased fat oxidation in the liver;
Increasing cholesterol intestinal efflux;
Reducing lipid digestion and ameliorating the intestinal system involved in lipid sensing and energy metabolism regulation, and
Ameliorating gut barrier function through a restoration of tight junction proteins in the duodenum and colon.

All these mechanisms were accompanied by a negligible change in gut microbiota composition.

## Supplementary material

For supplementary material accompanying this paper visit https://doi.org/10.1017/jns.2019.10. click here to view supplementary material
