# Supplementary material for: Coffee prevents fatty liver disease induced by a high-fat diet by modulating pathways of the gut–liver axis
Source: J Nutr Sci. 2019 Apr 22;8:e15. doi: 10.1017/jns.2019.10 (PMC6477661; doi:10.1017/jns.2019.10)
Supplement: Supplementary file 1 [file S2048679019000107sup001.docx]

**Supplementary material**

**Supplementary Table S1.** Composition of the diets used into the study (g/kg)

|  | SD^a^ | HFD^b^ |
| --- | --- | --- |
| Casein | 210.0 | 265.0 |
| L-Cystine | 3.0 | 4.0 |
| High Amylose Corn Starch | 500.0 | --- |
| Maltodextrin | 100.0 | 160.0 |
| Sucrose | 39.14 | 90.0 |
| Anhydrous Milk fat | 20.0 | --- |
| Lard | 20.0 | 310.0 |
| Soyabean Oil | 20.0 | 30.0 |
| Cellulose | 35.0 | 65.5 |
| Mineral Mix, AIN-93G-MX (94046) | 35.0 | 48.0 |
| Calcium Phosphate, dibasic | --- | 3.4 |
| Vitamin Mix, AIN-93-VX (94047) | 15.0 | 21.0 |
| Choline Bitartrate | 2.75 | 3.0 |
| TBHQ, antioxidant | 0.01 | --- |

^a^ Low glycaemic control diet (TD.120455; Envigo Teklad)

^b^ Adjusted calories diet (60/Fat) (TD.06414; Envigo Teklad)

**Supplementary Table S2.** Details of target and endogenous control

| Gene | Assay ID |
| --- | --- |
| Mouse Gapdh | Mn 03302249 _g1 |
| MouseTjp1 | Mn 00493699_m1 |
| Cldn1 | Mm00516701_m1 |
| Ocln | Mm00500912_m1 |
| Ffar1 | Mm00809442_s1 |
| Ffar3 | Mm02621638_s1 |
| Pyy | Mm00520716_g1 |
| ACOX1 | Mm01246831_m |
| Ppar alpha | Mn00440939_m1 |
| Abca1 | Mm00442646_m1 |
| Abcg1 | Mm00437390_m1 |


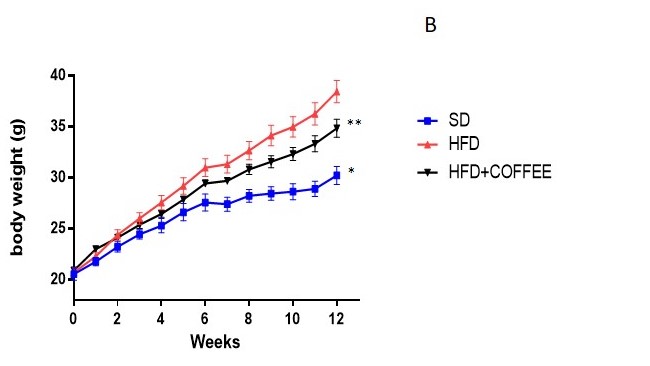

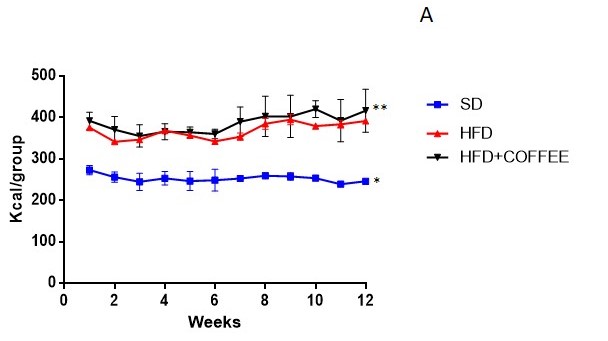


(A)

(B)

##

#

$$

$

**Supplementary Fig. S1*.*** Body weight (A) and food intake (B) time curves during the study period. In A) #p < 0.001 vs HFD and HFD+COFFEE, ##p< 0.001 vs HFD; in B) ^$^p < 0.0001 *vs* HFD and HFD+COFFEE, ^$$^p< 0.05 vs HFD; one-way ANOVA with Tukey’s post-test.


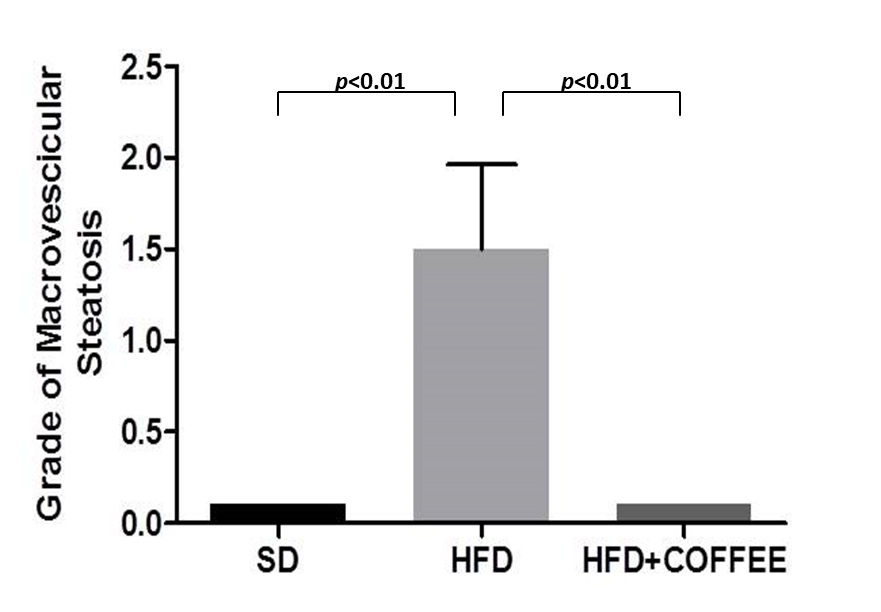


**Supplementary Fig. S2.** Grading of macrovesicular steatosis in livers from SD, HFD and HFD+COFFEE mice. The difference between HFD and the other groups was statistically significant (p<0.01).

**Lipid composition of caecal content**

For lipid extraction caecal material was diluted 1:10 in water, sample was vortexed for 1 min and 50 μL of fecal suspension were extracted using 1 mL of chloroform:methanol (2:1, v/v). Samples were vortexed for 20 s and centrifuged at 14800 rpm, 10°C, for 5 min. This operation was repeated twice, supernatants were united and 2 mL of water 0.07 M KCl were added to induce phase separation. Organic phase was dried under a stream of nitrogen and suspended in 500 µL of acetonitrile/isopropanol/water (65:30:5, v/v/v), ultracentrifuged at 14800 rpm for 3 min and analysed by LC-high resolution mass spectrometry (HRMS) analysis.

Results are presented in Supplementary Fig. S3.

**Supplementary Fig. S3.** Total lipids (A) and free fatty acids:triglycerides ratio (B) of the caecum content from SD (black), HFD (light grey) and HFD+COFFEE (grey) mice. Different letters on the bars indicate significant difference (Tukey’s t test)

Data showed that HFD+COFFEE mice had 2.2 and 2.5 times higher amount of total lipids in the caecum than HFD and SD mice, respectively. Interestingly, looking at the lipid composition, HFD+COFFEE mice showed a free fatty acids:triglycerides ratio that was ten times lower than HFD mice (0.02% vs 0.16%).


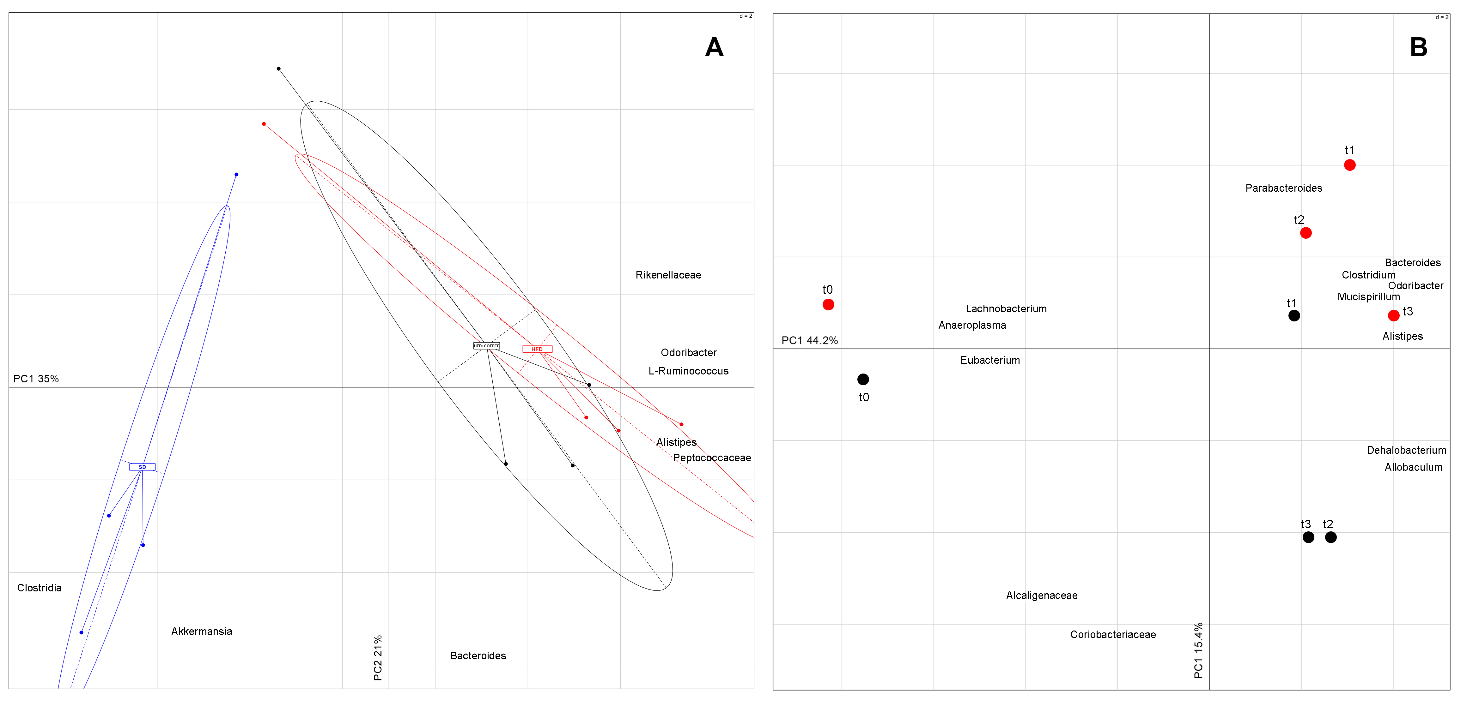


**Supplementary Fig. S4.** Principal component analysis (PCA) based on the genus level microbiota of all the samples analysed in this study (A) or only mice in HFD (B). The first two principal components were plotted. The centre of gravity for each cluster is marked by a rectangle indicating the sample group. Only the taxa with a loading score > 0.5 are shown in the figure. Samples are coloured according to the dietary treatment: blue, SD mice; red, HFD mice; black, HFD+COFFEE mice) and coded according to the length of treatment (t0, baseline; t1, 2 weeks; t2, 6 weeks; t3, 10 weeks).

Data showed that the gut microbiota of HFD mice was clearly different from SD (A). However, when considering only HFD mice (drinking coffee or not), a certain degree of separation was observed for mice treated with coffee for 6 and 10 weeks, that grouped apart from those not drinking coffee (B).
